# Gene Expression Profiling in Human High-Grade Astrocytomas

**DOI:** 10.1155/2011/245137

**Published:** 2011-08-07

**Authors:** Zhongyu Liu, Zhiqiang Yao, Chao Li, Yicheng Lu, Chunfang Gao

**Affiliations:** ^1^Anal-Colorectal Surgery Institute, No. 150 Central Hospital of PLA, Luoyang, Henan 471031, China; ^2^Department of Neurosurgery, No. 150 Central Hospital of PLA, Luoyang, Henan 471031, China; ^3^Division of Head and Neck Surgery, Sichuan Cancer Hospital, Chengdu 610041, China; ^4^Changzheng Hospital, Second Military Medical University, Shanghai 200003, China

## Abstract

Diffuse astrocytoma of (WHO grade II) has a tendency to progress spontaneously to anaplastic astrocytoma (WHO grade III) and/or glioblastoma (WHO grade IV). However, the molecular basis of astrocytoma progression is still poorly understood. In current study, an essential initial step toward this goal is the establishment of the taxonomy of tumors on the basis of their gene expression profiles. We have used gene expression profiling, unsupervised (hierarchal cluster (HCL) and principal component analysis (PCA)) and supervised (prediction analysis for microarrays (PAM)) learning methods, to demonstrate the presence of three distinct gene expression signatures of astrocytomas (ACMs), which correspond to diffuse or low-grade astrocytoma (WHO grade II), Anaplastic astrocytoma (WHO grade III) and Glioblastoma multiforme (WHO grade IV). We also demonstrate a 171 gene-based classifier that characterize the distinction between these pathologic/molecular subsets of astrocytomas. These results further define molecular subtypes of astrocytomas and may potentially be used to define potential targets and further refine stratification approaches for therapy. In addition, this study demonstrates that combining gene expression analysis with detailed annotated pathway and gene ontology (GO) category resources was applied to highly enriched normal and tumor population; it can yield an understanding of the critical biological mechanism of astrocytomas.

## 1. Introduction

Astrocytomas (ACMs) are cancers of the brain that originated in star-shaped brain cells called astrocytes; ACMs account for roughly 75% of neuroepithelial tumors. Of the numerous grading systems that have been devised, the most commonly used system for ACMs is the World Health Organization (WHO) grading system [1]. The WHO system assigns a grade from I to IV, with I being the least aggressive and IV being the most aggressive [2]. The number of collected ACM specimens is relatively low compared with other tumor collections, and there have been few reports of in-depth coverage using microarray technology. This study of ACM gene transcription profiles sets the stage for further discovery of the basic mechanisms that underlie the diseased state of ACM, which will help to identify targets for diagnosis and therapeutic intervention. The raw data has been deposited in the Gene Expression Omnibus (GEO; http://www.ncbi.nlm.nih.gov/projects/geo/) so that other researchers have access to it. Our analysis provides novel insights into differences between normal and malignant cancer tissues that may be used in assessing the impact of therapy on ACM cells. Because WHO grade I ACM often occurs in children, this study mainly focused on typically adult-onset ACM. The independent series of 15 ACMs consisted of five samples each of WHO grades II, III, and IV. In current study, an essential initial step toward this goal is the establishment of the taxonomy of tumors on the basis of their gene expression profiles. We aimed at identifying expression profiles that differentiate three groups of astrocytic glioma: WHO grades II, III, and IV. In a novel gene selection approach, we combined unsupervised statistical analysis with hierarchical clustering (HCL) [3], an supervised method called PAM [4] that applies nearest shrunken centroid analysis to identify correlated groups of genes that distinguish between the various tumor subtypes. Here, we demonstrate that ACMs can be separated according to their gene expression profiles. By using expression data of the most informative separating gene clusters, we were successful to construct an almost perfect tumor clustering model. Class prediction was performed by using prediction analysis of microarrays for excel package that applies nearest shrunken centroid analysis [4] to determine 37 predictor genes that achieve optimal prediction accuracy for sample classification. Meanwhile, we have also applied the gene sets to systems-level pathway analysis and identified molecular pathways and networks that are dysregulated between ACM and normal tissues. To build on this approach, functional enrichment analysis based on BioCarta and Kyoto Encyclopedia of Genes and Genomes (KEGG) pathways [5, 6] can be used to illustrate causal relationships between genes (gene products). While gene ontology (GO) [7] is organized into hierarchical annotations in the context of normal cellular function, the BioCarta and KEGG database organizes genes (gene products) into pathway reaction maps and functional complexes, including some disease-specific pathways. Our study provides valuable information for pharmaceutical screening programs and future cancer research.

## 2. Materials and Methods

The mean age of patients was 48.6 years old (range, 18–64). Fifteen human pathologic ACM specimens and corresponding normal brain tissue samples were obtained from resected tissues in the Neurosurgery Department of Shanghai Changzheng Hospital (Shanghai, China) between 2004 and 2006. The normal brain control samples were obtained as overlying cortex during routine resection of deep intercerebral metastases. All protocols and consent forms were approved by the hospital's institutional review board, and informed consent was obtained from all patients. The pathological diagnoses of the specimens were confirmed by two senior clinic-pathological experts, Fulin Zhang and Yichang Yu, at the Department of Pathology, Huashan Hospital, Fu Dan University (Shanghai, China) before RNA extraction was performed. Specimens were snap frozen in liquid nitrogen and stored at −80°C. Histological characteristics of tumor samples and clinical disease stage, five WHO grade II, III, and IV ACMs and one pooled and three additional normals, are shown in Table 1.

RNA was extracted using Trizol reagent and further purified using the Qiagen RNeasy Mini Kit according to the manufacturer's instructions. RNA quality was assessed by formaldehyde agarose gel electrophoresis and was quantified by spectrophotometry. The samples were hybridized to the Affymetrix Human Genome U133 Plus2.0 GeneChip arrays according to the Affymetrix protocols. The arrays were scanned with a GeneChip Scanner 3000. The scanned array images were processed with GeneChip Operating software (*GCOS v1.3*), and the CEL files were extracted for further analysis. The raw data were deposited in the Gene Expression Omnibus (GEO, http://www.ncbi.nlm.nih.gov/projects/geo/) with accession series number GSE19728, consisting of 17 different grades tumor samples plus four normal brain tissue sample, and GSE21354 including three additional normal brain tissue specimens the GEO sample names have been shown in Table 1.

The CEL files from all datasets (newly generated array data from 15 ACMs, including 5 WHO II, 5 III, and 5 IV and unmatched pooled normal and 3 additional normal samples) were imported into the statistical software R (v2.10.1.) [8] using Bioconductor (v2.5.11) [9]. After pre-installing two key packages, *hgu133plus2cdf* (v.2.5.0) and *hgu133plus2probe *(v.2.5.0), in R environments, the quality control of Affymetrix arrays (Affymetrix Hu133plus2.0) was first performed using the package* affy *(v 1.24.2) and *simpleaffy *(v 2.22.0) for Raw CEL files. RNA degradation was assessed using the function *AffyRNAdeg*() from the *affy* package. We checked the results of quality control implementation; all the qualities of arrays were pretty satisfied (see Supplementary Figure  1 in Supplementary Material available online at doi: 10.1155/2011/245137), then the raw data were normalized using the package *gcRMA* [10]. We used the “affinities” model of *gcRMA*, which uses mismatch probes as negative control probes to estimate the nonspecific binding of probe sequences. The normalized values of expression are in log2 scale, which attenuates the effect of outliers. The presence/absence calls provide a statistical measure of the presence of a transcript within the tested biological sample. Details of the calculations can be found in the Affymetrix Microarray Suite User Guide [11]. For the absolute detection of transcripts (presence or absence calls), the method we used [12] replaces all MM probe values by a threshold value which is based on the mean *PM* value (after gcRMA transformation) of probe sets that are very likely to have absent target transcripts. This removes the influence of probe sequence affinity and results in better performance than the MAS 5 algorithm [13]. In current study, the command *mas5calls*() of Bioconductor was used to generate MAS 5.0 *P*/*M*/*A* calls. Of the resulting output only the presence/absence calls were extracted and then mapped to the expression values output from the gcRMA [14]. We then saved the normalized expression values with the corresponding *P*/*M*/*A* values in a matrix. This matrix was regarded as data source in TIGR MeV Version 4-6-01 software tool (http://www.tm4.org/) [15]. For filter in MeV operation platform, (a) we first removed a set of probes that were used for quality control; (b) a matrix was composed of each probe ID, expression value, and its corresponding sample CALL (CALL: *P*, present; *A*, absent, *M*, medium); the following formula was used to filter the redundant probes,

(1) $$\text{value}=\frac{N\left(P\right)}{N\left(P\right)+N\left(A\right)+N\left(M\right)}\times 100\text{\%},$$

where *N*(*P*), *N*(*A*), *N*(*M*) stand for the number of *P*, *A*, *M*, respectively; if the value ≤10%, the corresponding probe ID would be excluded. Briefly, the probe with *P*/(*P* + *M* + *A*)% ≤ 10 was removed.

Analysis of variance (ANOVA) [16] is a technique that assesses whether a set of measurements from two or more experimental groups indicates, given observed variance, that the groups are different. For microarrays the measurements are the expression levels of one transcript, and the groups correspond to the experimental sample groups. The most basic type of ANOVA is a one-way ANOVA. In a one-way ANOVA, the sample groups are stratified along a single experimental variable. Currently, only one-way ANOVA is implemented in TIGR MeV (V 4-6-01) software tool. The user is initially required to enter the number of groups in our study, the numbers are set to 4 (ACMs II, III, IV, and normals). We then fit one-way ANOVA models with separate means for each of the four groups. Standard Bonferroni correction is calculated for each gene, and a gene is considered significant if *P* value associated with its Standard Bonferroni correction is smaller than the critical *P* value (0.01). Currently, *P* values are computed only from the Standard Bonferroni correction. We performed standard Bonferroni correction for each of the null hypotheses that the means of the average intensities of the four groups were equal; the alternative hypothesis was that at least one mean was different. After one-way ANOVA processing, the significance probe sets (saved as new original data source) were performed by HCL in TIGR MeV software tool and principal components analysis (PCA) [17] using the *R* function *prcomp *(…, scale = TRUE) of Bioconductor version 2.2. HCL was performed using the Euclidean distance and the average linkage algorithm.

For all kinds of data mining algorithms, we performed unsupervised data mining including HCL; on the other hand, for the supervised mining method, we chose prediction analysis of microarrays (PAM) [4] for class predication of all samples. The filtered dataset was separated in learning dataset including two normal samples, three WHO grade II, III, and IV ACMs, and test datasets consisting of two normal samples, two WHO grade II, III and IV ACMs using 10-fold cross-validation. One-way ANOVA was implemented to define the significant probes in TIGR MeV (V 4-6-01) software tool all the parameter settings and performances referred to the preceding descriptions. Class prediction with 10-fold cross validation using PAM for excel package was performed for the 996 significant probes exported from TIGR MeV. (http://www-stat.stanford.edu/~tibs/PAM/) (version 2.212) [4]. In PAM operation, with threshold (Δ) of 41.0, we built a classifier containing 171 probes which gave “zero” misclassification error (refer to Supplementary Figure  2); the classifier generated was applied to the test data which is matrix data consisting of aforementioned 996 significant probes and corresponding expression values in the test datasets.

The differentially expressed genes (DEGs) output from one-way ANOVA performance. All DEGs were first distributed for functional profiles using Gene Ontology terms including biological process, cellular component, and molecular function (http://david.abcc.ncifcrf.gov/) [18], with hypergeometric distribution [19] and FDR correction parameter settings [20]. The three category-annotated files containing the gene annotations and categories of biological process, cellular component, and molecular function, were exported using DAVID online tools [20]. In each file, the GO terms in each annotated category with corrected *P* values (expanded) <0.05 were collected. Unannotated genes were excluded. The GO database was updated as of July 1st, 2009. Two databases, BioCarta and KEGG, were used to identify significantly altered pathways. Dys-regulated pathways were identified using the DAVID system [18, 21] by means of mapping the DEGs generated by one-way ANOVA into the BioCarta and KEGG databases. A *P* value for each pathway was obtained using the hypergeometric test described by Zhang et al. [22].

## 3. Results

We analyzed the expression of 54,676 probe IDs using Affymetrix HU-133PLUS 2.0 GeneChip microarrays for 15 ACM tissue samples consisting of five each of grade II, III, and IV tumors and four normal tissue specimens. The quality control was implemented for the raw data after normalization. Basic quality control for Affymetrix array consists of checking for RNA degradation and examining the expression for control genes, scaling factors, percentage of present genes, and the average background. Supplementary Figures  1(a) and  1(b) show the general quality control statistics and RNA degradation of all arrays; the information in Supplementary Figure  1 explicitly illustrates that the quality of all those arrays is completely acceptable.

### 3.1. HCL and PCA

**Figure 1:**
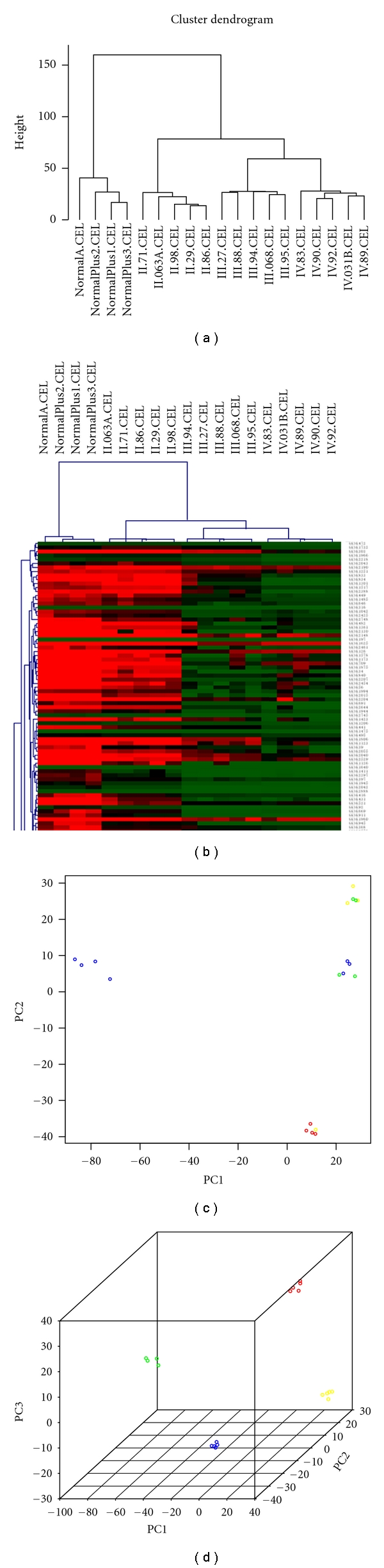
The exhibition of results of HCL and PCA. Hierarchal clustering: the analysis was performed using the Euclidean distance and the average linkage algorithm; principal component analysis with scaling. (a) dendrogram plot: the astrocytomas tissue group and normal-tissue group were separated obviously in the top node; tumor node is composed of two branches, one is just WHO II grade, and another includes WHO III cluster and WHO IV cluster; (b) heatmap plot: each column represents the expression levels for all genes in a particular sample, whereas each row represents the relative expression of a particular gene across all samples. The expression level of any given gene in any given sample (relative to the mean expression level of that gene across all tissue samples) is reported along a color scale in which red represents transcriptional upregulation, green represents downregulation, and the color intensity indicates the magnitude of deviation from the mean; (c) the two main principal components 2D-plot; (d) the three main principal components 3D-plot. In Figures 1(c) and 1(d): different colors stand for different pathological stages, Green, blue, yellow, and red represent normal, II, III, and IV grade, respectively.

After gcRMA normalization and filters, the remaining 32,095 probes were performed using one-way ANOVA, and then the 4015 probes left were executed by PCA, HCL, and class prediction (see Methods and Materials). The dendrogram and heatmap (Figures 1(a) and 1(b), resp.) from hierarchal clustering and two (2D plot Figure 1(c)) and three (3D plot Figure 1(d)) main principal components from PCA analysis illustrate that ACMs and normal tissue samples can be almost perfectly separated according to their pathological stages.

We used a HCL and PCA algorithm to study the changes in the ACMs on a genome-wide level. The resultant data are shown in Figure 1. In Figure 1(b), each row represents the expression levels of a particular gene across all samples, and each column represents the expression level of all of the genes tested for each sample. At a glance, our genome-wide level analysis showed that the ACM samples were grouped into one cluster, whereas the normal control samples were clustered separately. This shows that genome-wide transcript profiling can be used to distinguish ACM from normal tissue. Furthermore, within the cancer cluster, tumors from a particular pathologic grade or clinical stage were also clustered reliably with other tumors of the same grade or stage, indicating that there is significant molecular profile among tumors classified within a particular pathologic grade or clinical stage (Figures 1(a) and 1(b)). The tumor-tissue cluster consists of two subbranches, one is only WHO grade II cluster and another includes WHO III and WHO IV clusters. Multidimensional scaling using the first two (Figure 1(c)) and three (Figure 1(d)) principal components, a linear projection method that reduces the complex dimensionality of microarray data to create a three-dimensional plot that visualizes the relatedness of the tumors, was then used to test whether the above subsets could be used to distinguish WHO II, III, and IV tumor and normal brain. This analysis showed a clear separation of all three groups based on these genes. In general, the analysis of HCL and PCA (Figure 1) explicitly indicates that classification of ACM on the basis of genome-wide level is completely consistent with clinical stages, which is an inspiring result.

### 3.2. ACM Classification

**Figure 2:**
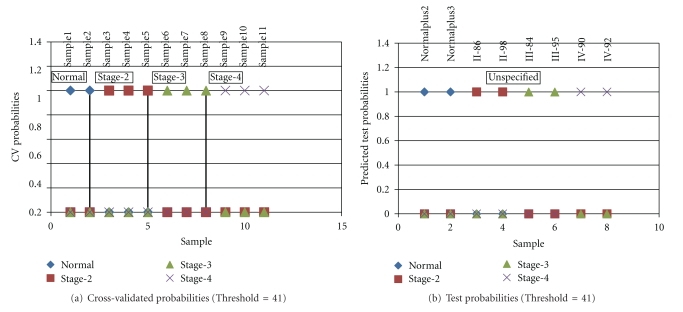
Estimated probabilities for the cross-validation (a) and test data (b). Samples are partitioned by the true class (a) and the predicted class (b). All 11 of the training samples and all 8 of the test samples known to be astrocytomas are correctly classified.

We applied a supervised analysis using “nearest shrunken centroid classifier” and the PAM for excel package (version 2.212). As Supplementary Figure  2 shows, the threshold (Δ) of 41.0 was chosen as arguments, we built a classifier containing 171 genes which gave misclassification error  =  0 (Supplementary Figure  2). We then used this classifier to predict the subtypes of the 6 tumors and 2 normal samples analyzed in this study. As Table 2 and Figure 2 show, the results of predication are in agreement with clinical pathological classification. In order to gain the marker genes of different pathological grade of ACM, we further run *listgenes *button* with threshold = 41.0 *in PAM for excel package. As a result, 12 genes for grade II, 12 genes for grade III, and 13 genes for grade IV (Table 3) were selected as markers to distinguish different grade pathological tissues, excluding the 132 genes referred to as normal tissue markers.

### 3.3. GO Categories

Similarly, after gcRMA normalization and filtering and one-way ANOVA procedures, the 4015 probes left were mapped to 2649 unique gene symbols, and those genes were further analyzed using DAVID system (parameter settings, refer to Methods and Materials) (http://david.abcc.ncifcrf.gov/). The significantly overrepresented GO terms (corrected *P* values < 0.5 and no gene >10) are shown in Supplementary Table  1. In the cellular component category, 19 GO terms were significant, including extrinsic to membrane, endomembrane system, cell junction, nuclear lumen, chromatin remodeling complex, integral to organelle membrane, intrinsic to organelle membrane, and plasma membrane part. Most of these were enriched in the “core dataset” membrane at the same significance level. In the molecular function category, 17 GO terms were enriched, including enzyme binding, metal ion binding, zinc ion binding, cation binding, Ras GTPase binding, transition metal ion binding. The predominant terms related to biological processes consisted of intracellular signaling cascade, positive regulation of apoptosis, and positive regulation of programmed cell death.

### 3.4. Dysregulated Pathways

**Figure 3:**
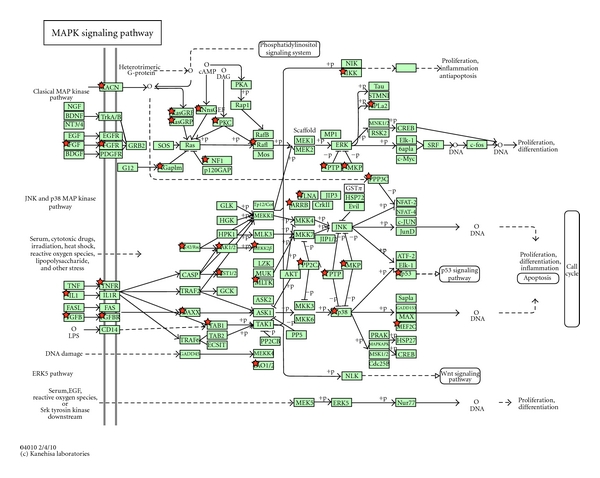
MAPK signaling pathway. Red stars marks indicate dysregulated genes.

The significance of enrichment from one-way ANOVA implementation was calculated by the hypergeometric test with microarray type Affymetrix HU-U133-PLUS-2. 19 dysregulated pathways with more than five DEGs and with *P* < 0.01 in Biocarta database and in the KEGG data base were identified using DAVID (Supplementary Table  2). The five most significant BioCarta pathways were first multivalent nuclear factor, FAS signaling pathway (CD95), p38 MAPK signaling pathway, control of skeletal myogenesis by HDAC and calcium/calmodulin-dependent kinase (CaMK), role of BRCA1, BRCA2, and ATR in cancer susceptibility. The top five significant dysregulated pathways from the KEGG database were pathways in cancer, mitogen-activated protein kinase (MAPK) signaling pathway (Figure 3), inositol phosphate metabolism, dilated cardiomyopathy, focal adhesion, calcium signaling pathway.

## 4. Discussion

Although microarray technology is now available to many researchers, methods for evaluation and interpretation of microarray data are still evolving. To date, most microarray studies presented from clinical settings concentrated on classification and/or pattern recognition for discrimination among different tumor types or subgroups. Here, we generated expression profiles of 15 tumors using HG-U133plus2.0 chips (Affymetrix) to characterize the astrocytomas transcriptome comprising the clinical stages. We focused on molecular definition of clinical and biological subgroups and the identification of molecular transcription-level gene signatures of astrocytomas.

We used a HCL and PCA algorithm to study the changes in gene expression in the ACMs on a genome-wide level. Consequently, our analysis showed a clear separation of all three groups based on these genes, which suggest that there are classable differences underlying transcriptional level of genes amongst tissue-type categorization of astrocytomas. Fortunately, we are successful to classify accurately the ACMs into three subgroups, WHO II, III, IV, by PAM method and detect the marker genes aiming at the different pathological stages of ACM (Table 3). It is interesting to compare our list of 37 genes with genes that are presently considered to be diagnostic for ACMs. In the future research, several gene products will be measured by immunostaining and PCR to distinguish he ACMs from each other: 12 upregulated genes including gene C12orf39 are specific for WHO II; 8 upregulated genes and 4 downregulated genes are specific for WHO III, and 13 upregulated genes are specific for WHO IV. The data are displayed in Table 3 to demonstrate their shortcomings in comparison to the genes identified by nearest shrunken centroids.

We further analyzed the gene expression profiling of ACMs using GO category and pathway using Biocarta and KEGG database. The dys-regulated pathways and GO terms implicated in ACMs were reported. In the dys-regulated pathways implicated in ACM, inositol phosphate metabolism was reported to be involved in astrocytomas [23]. Wang et al. [24] demonstrated that p125 focal adhesion kinase (p125FAK) is a cytoplasmic tyrosine kinase that is activated upon engagement of integrin cell adhesion receptors and initiates several signaling events that modulate cell function in vitro [24]. A recent report suggested that the communication pathway has received utmost attention since it is known that astrocytic calcium signals can be induced by neuronal stimulation and can be communicated back to the neurons to modulate synaptic transmission [25] The current study represents the first effort to simultaneously compare the transcriptional profiles of highly enriched ACMs and normal tissues from a variety of patients using modern microarray technology. The comparison of expression patterns of ACM and normal tissues enhanced our ability to identify genes and pathways that are disrupted in ACM tumor tissue. This analysis provides critical insights into the differences between normal and malignant cancer tissue populations that may be used for assessing therapies targeting ACM.

Analysis of specific cellular processes and pathways within the different transgenerationally altered gene sets is shown in Supplementary Table  2. The pathways containing the highest numbers of affected genes are listed. The mitogen-activated protein kinase (MAPK) signaling pathway was affected in all of the altered gene sets (Figure 2). MAPK signaling is responsive to a large number of regulatory factors [26]; MAPK signaling pathways are evolutionarily conserved in eukaryotes and are involved in many cellular processes, including growth, differentiation, apoptosis, and the immune response [27]. These pathways feature a conserved signaling cascade downstream of small GTPases of the Ras and Rho families. The cascade consists of a MAPK kinase kinase kinase, which phosphorylates and activates a MAPK kinase kinase, which then activates the MAPK by phosphorylation on Thr and Tyr residues within a conserved motif located in the kinase activation loop [27]. A considerable number of studies have demonstrated the dysregulation of MAPK signaling in a variety of cancers [28]. For example, Bakin et al. [29] showed that MAP kinase activation correlates with the progression to advanced hormone refractory disease in patient samples and that stable expression of Ras effector loop mutants that activate the Ras/MAP kinase pathway is sufficient to reduce the androgen requirement of LNCaP prostate cancer cells for growth, prostate-specific antigen expression, and tumorigenicity [29]. A recent report by Zafon and Obiols [30] showed that constitutive activation of the MAPK signaling pathway is a major event in the progression of papillary thyroid carcinoma [30]. Mizoguchi et al. [31] found that MAPK signaling was correlated with malignant astrocytic gliomas [31]. The results of our analysis suggest that this pathway is dysregulated in ACM and may contribute to the pathogenesis of ACM.

## 5. Conclusion

There are an abundance of data on the gene expression profiles of normal and ACM tissue. A few studies have recently been published that aimed to detect molecular markers to distinguish the different grade ACMs. In our study, the method of nearest shrunken centroids was successful in finding genes that accurately predict classes. The method found a set of 37 genes that was able to assign ACMs to one of three classes, WHO II, III, and IV ACMs, with 100% accuracy. The success of our methodology has implications for improving the diagnosis of cancer. The method efficiently finds and ranks genes that can distinguish one type of tumor from another. Ultimately, it may be used to search for genes that are predictive for response to chemotherapy. For ACMs, the predictive genes are attractive candidates for raising antibodies suitable for immunostaining. Immunohistochemistry has an advantage for analyzing difficult specimens, because it allows the pathologist to localize the stain to tumor cells. In addition, our results suggest that RNA-based diagnostic tests may soon become feasible, based on either small-scale microarrays or quantitative PCR.

In addition, we performed clustering and classification of GO categories and dysregulated pathways on the basis of gene expression in ACM versus normal brain tissue. According to the microarray-wide gene level, we first reported that categorization of ACMs by gene expression using HCL, PCA, and PAM is almost completely in agreement with clinical pathological stages. This study sets the stage for the further discovery of basic mechanisms that underlie a diseased state of astrocytoma and will be useful in targeting genes for diagnosis and therapeutic intervention.

Comparing the expression signatures of ACM from different grades (II, III, and IV) of ACM patients to that of normal tissue, we identified the DEGs of ACMs, on the basis of which, GO terms and dysregulated pathways implicated in astrocytomas have been identified and profiled. Supplementary Table  2 summarizes the top dysregulated pathways by *P* value from the KEGG and BioCarta databases and shows the number of genes referenced by the KEGG and Biocarta pathways and the numbers of genes that were found in our data set by one-way ANOVA analysis. For each KEGG or Biocarta pathway, an unbiased systems-level pathway analysis was implemented by DAVID. The *P* value of each pathway reflects the significance of pathway dysregulation. These pathways, such as MAPK signaling, were identified as significant. The DEGs were effectively categorized by GO terms between ACM and normal tissue samples.

The use of pathway analysis and GO categories to derive gene signature networks has several advantages. First, it allows a transition from biological function at the molecular level to a more global, systems approach to disease and biological processes. Second, it identifies key regulators and transcription factors that might not be identified by microarray technology alone. Third, it permits further interpretation of gene expression data by providing information on protein-protein interactions, metabolic, signaling, and transcription regulatory networks. This paper thus provides a novel global view of the differences between ACMs and normal tissue at the transcriptional level. Further investigation and validation by experiments should be targeted to the processes that have been identified to play key roles in metabolic and signaling pathways in ACM. The raw data has been deposited in GEO (http://www.ncbi.nlm.nih.gov/projects/geo/) so that other researchers can have access to this important resource.

## Supplementary Material

Supplementary Figure 1: The quality control status map. Quality control plot for all arrays. A: Quality control statistic plot. B: RNA degradation plot.
Supplementary Figure 2: ACMs classification.
Supplementary Table 1: Significant overrepresented GO terms for the set of differentially expressed genes of ACM tissue.
Supplementary Table 2: Dys regulated pathways (*P* values < 0.01) associated with ACM were identified by DAVID.

## Figures and Tables

**Table 1:** Patients' characteristics.

No	Array ID	GEO No	Title	Histological diagnosis	Age/Gender	WHO grade
1	190-063A	GSM492652	Astrocytomas_T2-1-Signal	Diffuse or low-grade astrocytoma	72, M	II
2	0286-29	GSM492653	Astrocytomas_T2-2-Signal	Diffuse or low-grade astrocytoma	32, M	II
3	0286-71	GSM492654	Astrocytomas_T2-3-Signal	Diffuse or low-grade astrocytoma	73, M	II
4	0286-98	GSM492655	Astrocytomas_T2-4-Signal	Diffuse or low-grade astrocytoma	45, M	II
5	0286-86	GSM492656	Astrocytomas_T2-5-Signal	Diffuse or low-grade astrocytoma	65, M	II
6	0286-94	GSM492657	Astrocytomas_T3-1-Signal	Anaplastic astrocytoma	18, F	III
7	0286-95	GSM492658	Astrocytomas_T3-2-Signal	Anaplastic astrocytoma	64, F	III
8	0190-068	GSM492659	Astrocytomas_T3-3-Signal	Anaplastic astrocytoma	28, M	III
9	0286-27	GSM492660	Astrocytomas_T3-4-Signal	Anaplastic astrocytoma	45, M	III
10	0286-88	GSM492661	Astrocytomas_T3-5-Signal	Anaplastic astrocytoma	54, M	III
11	0190-031B	GSM492662	Astrocytomas_T4-1-Signal	Glioblastoma multiforme (GBM)	68, M	IV
12	0286-83	GSM492663	Astrocytomas_T4-2-Signal	Glioblastoma multiforme (GBM)	62, M	IV
13	0286-89	GSM492664	Astrocytomas_T4-3-Signal	Glioblastoma multiforme (GBM)	80, M	IV
14	0286-92	GSM492665	Astrocytomas_T4-4-Signal	Glioblastoma multiforme (GBM)	78, F	IV
15	0286-90	GSM492666	Astrocytomas_T4-5-Signal	Glioblastoma multiforme (GBM)	69, M	IV
16	Normal Plus1	GSM525014	Normal	Normal brain tissues		Normal
17	Normal Plus2	GSM525015	Normal	Normal brain tissues		Normal
18	Normal Plus3	GSM525016	Normal	Normal brain tissues		Normal
19	0190-normalA	GSM492649	Normal	Pooled normal tissues		Normal

**Table 2:** Actual and predicted classification.

Sample labels	Normal plus2	Normal plus3	II-86	II-98	III-84	III-95	IV-90	IV-92
Predicted class labels	Normal	Normal	Stage-2	Stage-2	Stage-3	Stage-3	Stage-4	Stage-4
Normal	1	1	0	0	0	0	0	0
Stage-2	0	0	1	1	0	0	0	0
Stage-3	0	0	0	0	1	1	0	0
Stage-4	0	0	0	0	0	0	1	1

**Table 3:** The marker genes of three different grades of ACM.

Gene symbol	II-astro score	III-astro score	IV-astro score	Normal-score	II-mean (log base 2)	III-mean (log base 2)	IV-mean (log base 2)	Normal mean (log base 2)
EPHB1	15.4462	0	0	0	**6.869894**	2.55495	2.65409	2.72811
LOC732229	13.8658	0	0	0	**5.460512**	2.59371	2.77408	2.26366
C12orf39	8.5766	0	0	0	**5.361751**	2.238085	2.58682	2.48695
MARVELD3	7.8886	0	0	0	**6.800398**	2.37729	2.86839	2.39552
LOC283677	4.4758	0	0	0	**3.883122**	2.826663	2.71392	2.8898
C20orf42	3.5577	0	0	0	**7.627308**	2.859789	2.4558	2.52664
SPTLC3	3.2679	0	0	0	**4.01698**	2.255256	2.30132	2.59188
KCNQ1OT1	2.9128	0	0	0	**4.103731**	2.338234	2.2464	2.26363
FLJ21062	2.1167	0	0	0	**4.753168**	2.238085	2.48495	2.26875
ATP13A5	1.9707	0	0	0	**3.214755**	2.238085	2.2464	2.43345
PDXDC1	1.3847	0	0	0	**3.134729**	2.25661	2.26636	2.26363
CNNM2	0.8081	0	0	0	**3.148803**	2.27347	2.6133	2.54342

ZBTB39	0	15.0318	0	0	4.173501	**6.19055**	4.6856	4.68794
C6orf89	0	11.2219	0	0	2.621672	**4.699428**	2.91788	2.30613
LOC116236	0	11.0126	0	0	2.254329	**4.397317**	2.72612	2.44326
FKHL18	0	10.2107	0	0	2.258635	**3.994112**	2.2464	2.6121
CTRL	0	10.0666	0	0	2.526428	**3.937424**	2.31851	2.58541
TIRAP	0	9.5523	0	0	2.324936	**4.216794**	2.2464	2.75954
NEUROG1	0	2.5236	0	0	2.258056	**3.768388**	2.64751	2.27475
TMEM44	0	0.4603	0	0	2.315798	**3.374982**	2.45605	2.51802
RPUSD1	0	−0.647	0	0	4.034009	*2.703442*	3.84208	4.14466
DHRS7	0	−2.0711	0	0	3.425335	*2.573317*	3.60534	4.03533
AKAP7	0	−2.6126	0	0	4.723465	*2.652648*	5.11056	3.7332
PDE3B	0	−4.2169	0	0	5.989233	*4.535432*	6.10718	6.31594

C20orf160	0	0	14.9825	0	2.523126	**2.790445**	4.41487	2.27477
PKMYT1	0	0	11.89	0	2.644079	**2.486864**	3.84824	2.26751
TRPV2	0	0	10.5089	0	2.397686	**2.732524**	4.5048	3.31894
LEFTY2	0	0	5.3507	0	2.231583	**2.258491**	5.56688	2.28191
C20orf77	0	0	5.1848	0	2.333998	**2.404051**	3.48131	2.3379
MOBKL2C	0	0	3.8936	0	2.517987	**2.58549**	3.86723	2.45653
STAC	0	0	2.9965	0	2.275399	**2.238151**	6.32448	2.78522
GGA3	0	0	2.8351	0	2.295872	**2.433914**	3.46887	2.26651
VAV1	0	0	2.1584	0	2.396392	**2.515654**	4.53627	2.34353
TXNRD2	0	0	1.3886	0	2.582906	**2.358745**	3.38602	2.41848
SP100	0	0	0.9134	0	2.385938	**2.339246**	3.58699	2.37374
LCP2	0	0	0.7656	0	2.268675	**2.292975**	3.62601	2.88405
RHEB	0	0	0.6002	0	2.254732	**2.244571**	3.16583	2.31595

The first column lists the marker genes for different ACM WHO grades; the scores from 2th to 5th columns suggest the estimates of the class probabilities by PAM [4], which are similar to that used in linear discriminant analysis (LDA); the means from 6th to 9th columns represent the average values of log (base 2) gene expression. Bold indicates a marker gene of one WHO grade ACM relative to other WHO grades which are overexpressional; italic indicates those that are underexpressional.

## References

[1] A free encyclopedia to glossary on the Internet: Citation formats. http://en.wikipedia.org/wiki/Astrocytoma.

[2] Scott JN, Rewcastle NB, Brasher PMA (1998). Long-term glioblastoma multiforme survivors: a population-based study. *Canadian Journal of Neurological Sciences*.

[3] Everitt B (1974). *Cluster Analysis*.

[4] Tibshirani R, Hastie T, Narasimhan B, Chu G (2002). Diagnosis of multiple cancer types by shrunken centroids of gene expression. *Proceedings of the National Academy of Sciences of the United States of America*.

[5] Kanehisa M (1997). A database for post-genome analysis. *Trends in Genetics*.

[6] Kanehisa M, Goto S (2000). KEGG: Kyoto encyclopedia of genes and genomes. *Nucleic Acids Research*.

[7] Ashburner M, Ball CA, Blake JA (2000). Gene ontology: tool for the unification of biology. *Nature Genetics*.

[8] R_Development_Core_Team R: A language and environment for statistical computing.

[9] Gentleman RC, Carey VJ, Bates DM (2004). Bioconductor: open software development for computational biology and bioinformatics. *Genome Biology*.

[10] Wu Z, Irizarry RA (2005). Stochastic models inspired by hybridization theory for short oligonucleotide arrays. *Journal of Computational Biology*.

[11] Affymetrix Affymetrix Microarray Suite User Guide.

[12] Schuster EF, Blanc E, Partridge L, Thornton JM (2007). Correcting for sequence biases in present/absent calls. *Genome Biology*.

[13] Roux J, Robinson-Rechavi M (2008). Developmental constraints on vertebrate genome evolution. *PLoS Genetics*.

[14] McClintick JN, Edenberg HJ (2006). Effects of filtering by Present call on analysis of microarray experiments. *BMC Bioinformatics*.

[15] Saeed AI, Sharov V, White J (2003). TM4: a free, open-source system for microarray data management and analysis. *BioTechniques*.

[16] Zar JH (1999). *Biostatistical Analysis*.

[17] Wu Z, Irizarry RA (2005). Stochastic models inspired by hybridization theory for short oligonucleotide arrays. *Journal of Computational Biology*.

[18] Huang DW, Sherman BT, Lempicki RA (2009). Systematic and integrative analysis of large gene lists using DAVID bioinformatics resources. *Nature Protocols*.

[19] Drǎghici S, Khatri P, Martins RP, Ostermeier GC, Krawetz SA (2003). Global functional profiling of gene expression. *Genomics*.

[20] Benjamini Y, Yekutieli D (2001). The control of the false discovery rate in multiple testing under dependency. *Annals of Statistics*.

[21] Dennis G, Sherman BT, Hosack DA (2003). DAVID: database for annotation, visualization, and integrated discovery. *Genome Biology*.

[22] Zhang B, Kirov S, Snoddy J (2005). WebGestalt: an integrated system for exploring gene sets in various biological contexts. *Nucleic Acids Research*.

[23] Cadman ED, Witte DG, Lee CM (1994). Regulation of the release of interleukin-6 from human astrocytoma cells. *Journal of Neurochemistry*.

[24] Wang D, Grammer JR, Cobbs CS (2000). P125 focal adhesion kinase promotes malignant astrocytoma cell proliferation in vivo. *Journal of Cell Science*.

[25] Braet K, Cabooter L, Paemeleire K, Leybaert L (2004). Calcium signal communication in the central nervous system. *Biology of the Cell*.

[26] Skinner MK, Anway MD, Savenkova MI, Gore AC, Crews D (2008). Transgenerational epigenetic programming of the brain transcriptome and anxiety behavior. *PLoS One*.

[27] Chang L, Karin M (2001). Mammalian MAP kinase signalling cascades. *Nature*.

[28] Dhillon AS, Hagan S, Rath O, Kolch W (2007). MAP kinase signalling pathways in cancer. *Oncogene*.

[29] Bakin RE, Gioeli D, Sikes RA, Bissonette EA, Weber MJ (2003). Constitutive activation of the Ras/mitogen-activated protein kinase signaling pathway promotes androgen hypersensitivity in LNCaP prostate cancer cells. *Cancer Research*.

[30] Zafon C, Obiols G (2009). The mitogen-activated protein kinase (MAPK) signaling pathway in papillary thyroid cancer. From the molecular bases to clinical practice. *Endocrinologia y Nutricion*.

[31] Mizoguchi M, Betensky RA, Batchelor TT, Bernay DC, Louis DN, Nutt CL (2006). Activation of STAT3, MAPK, and AKT in malignant astrocytic gliomas: correlation with EGFR status, tumor grade, and survival. *Journal of Neuropathology and Experimental Neurology*.

