# Supplementary material for: Gene Expression Profiling in Human High-Grade Astrocytomas
Source: Comp Funct Genomics. 2011 Aug 7;2011:245137. doi: 10.1155/2011/245137 (PMC3151497; doi:10.1155/2011/245137)
Supplement: Supplementary file 1 — Supplementary Figure 1: The quality control status map. Quality control plot for all arrays. A: Quality control statistic plot. B: RNA degradation plot. Supplementary Figure 2: ACMs classification. Supplementary Table 1: Significant overrepresented GO terms for the set of differentially expressed genes of ACM tissue. Supplementary Table 2: Dys regulated pathways (P values < 0.01) associated with ACM were identified by DAVID. [file 245137.f1.pdf]

Supporting Online Material for

Gene Expression Profiling in Human High-Grade Astrocytomas

Zhongyu Liu,[1] Zhiqiang Yao,[2] Chao Li,[3] Yicheng Lu,[* 4] and Chunfang Gao[*1]
1 Anal-Colorectal Surgery Institute, No. 150 Central Hospital of PLA, Luoyang, Henan 471031, China
2 Department of Neurosurgery , No. 150 Central Hospital of PLA, Luoyang, Henan 471031, China
3 Division of Head and Neck Surgery, Sichuan Cancer Hospital, Chengdu 610041, China
4 Changzheng Hospital, Second Military Medical University, Shanghai 200003, China
*Correspondence should be addressed to Chunfang Gao and Yichang Lu, gaochunfang2010@163.com

This PDF file includes:
Supplementary Figure 1
Supplementary Figure 2
Supplementary Table 1
Supplementary Table 2

Figures and legends

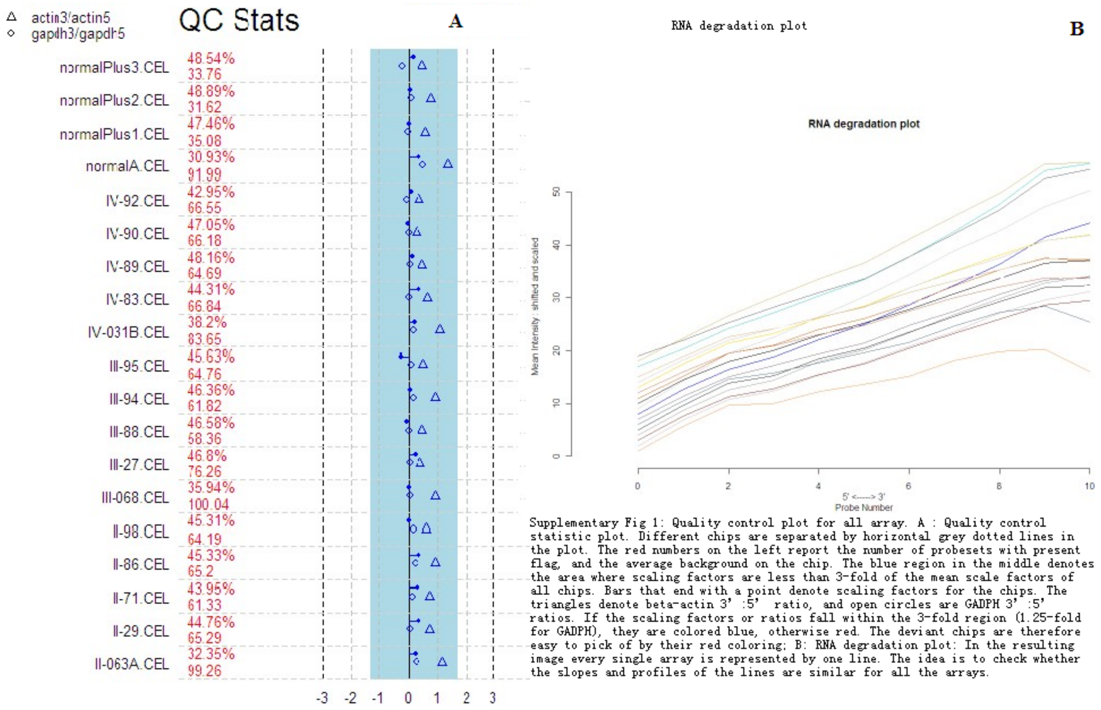

Supplementary Figure 1: The quality control status map. Quality control plot for all arrays. A: Quality control statistic plot. Different chips are separated by horizontal grey dotted lines in the plot. The red numbers on the left report the number of probe sets with present flag, and the average background on the chip. The blue region in the middle denotes the area where scaling factors are less than 3-fold of the mean scale factors of all chips. Bars that end with a point denote scaling factor for the chips. The triangles denote beta-actin 3’: 5’ ratio, and open circles are GADPH 3’: 5’ ratios. If the scaling factors or ratios fall within the 3-fold region (1.25-fold for GADPH), they are colored blue, otherwise red. The deviant chips are therefore easy to pick of by their red coloring: B: RNA degradation plot: In the resulting image every single array is

represented by one line. The idea is to check whether the slopes and profiles of the lines are similar for all arrays.

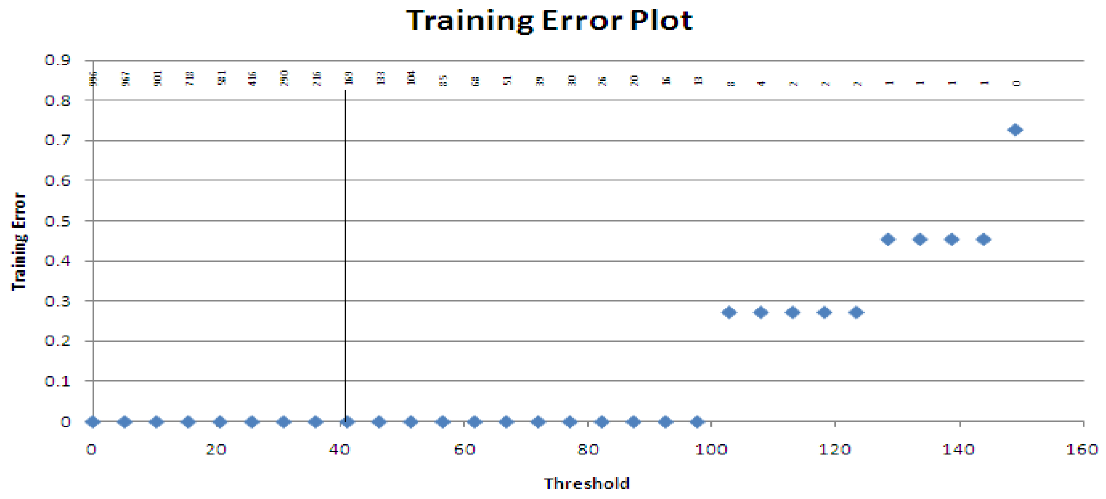

Supplementary Figure 2: ACMs classification: Training error plot and misclassification errors are shown as a function of the threshold parameter τ . The value τ (threshold)=41.0 is chosen and yields a subset of 171 selected genes, including the 132 genes referred to as normal tissue markers.

Tables and legends

Supplementary Table 1: significant overrepresented GO terms for the set of differentially expressed genes of ACM tissue. 2649 differentially expressional genes were analyzed using DAVID system (parameter settings, refer to Methods and Materials) (http://david.abcc.ncifcrf.gov/). The significantly over-represented GO terms (FDR< 0.5 and Number of genes within a single pathway >10) are identified.

GO Ontology	Term	Count	FDR
biological process	GO:0007242~intracellular signaling cascade	197	0.018959
	GO:0043065~positive regulation of apoptosis	79	0.0973
	GO:0043068~positive regulation of programmed cell death	79	0.124233

GO:0010942~positive regulation of cell death	79	0.145894
GO:0016568~chromatin modification	54	0.295214
GO:0008219~cell death	117	0.352727
GO:0007010~cytoskeleton organization	77	0.451875
GO:0016265~death	117	0.461443
GO:0015674~di-, tri-valent inorganic cation transport	38	0.526657
GO:0008104~protein localization	138	0.534804
GO:0006816~calcium ion transport	32	0.80372
GO:0006350~transcription	294	0.894109
GO:0042981~regulation of apoptosis	126	0.952536
GO:0010627~regulation of protein kinase cascade	48	1.184242
GO:0000165~MAPKKK cascade	38	1.28682
GO:0043067~regulation of programmed cell death	126	1.404968
GO:0010941~regulation of cell death	126	1.610926
GO:0007254~JNK cascade	17	1.769651
GO:0033500~carbohydrate homeostasis	15	2.074711
GO:0042593~glucose homeostasis	15	2.074711
GO:0045184~establishment of protein localization	119	2.203149
GO:0015031~protein transport	118	2.248817
GO:0006917~induction of apoptosis	57	2.546204
GO:0007243~protein kinase cascade	64	2.656027
GO:0012502~induction of programmed cell death	57	2.738234
GO:0033554~cellular response to stress	91	2.808061
GO:0012501~programmed cell death	97	2.915256
GO:0006468~protein amino acid phosphorylation	104	3.515993
GO:0031098~stress-activated protein kinase signaling pathway	17	3.721198
GO:0006915~apoptosis	95	4.027922
GO:0051276~chromosome organization	79	4.088372

	GO:0030155~regulation of cell adhesion	29	4.183801
	GO:0045930~negative regulation of mitotic cell cycle	9	4.519829
	GO:0033674~positive regulation of kinase activity	43	4.602038
	GO:0007162~negative regulation of cell adhesion	13	5.611682
	GO:0007162~negative regulation of cell adhesion	13	5.611682
Cellular Component	GO:0019898~extrinsic to membrane	93	0.001248
	GO:0012505~endomembrane system	129	0.01277
	GO:0030054~cell junction	91	0.030208
	GO:0031981~nuclear lumen	211	0.091487
	GO:0016585~chromatin remodeling complex	21	0.121437
	GO:0044456~synapse part	49	0.16497
	GO:0005654~nucleoplasm	136	0.185168
	GO:0016605~PML body	13	0.231965
	GO:0031300~intrinsic to organelle membrane	33	0.256589
	GO:0045202~synapse	64	0.282216
	GO:0031301~integral to organelle membrane	29	0.327755
	GO:0044459~plasma membrane part	299	0.407759
	GO:0000118~histone deacetylase complex	14	0.596212
	GO:0031090~organelle membrane	160	0.705946
	GO:0005626~insoluble fraction	126	1.038663
	GO:0005624~membrane fraction	122	1.092698
	GO:0034703~cation channel complex	29	1.099605
	GO:0048471~perinuclear region of cytoplasm	52	1.159989
	GO:0044451~nucleoplasm part	88	1.463588
	GO:0005856~cytoskeleton	193	1.671759
	GO:0008021~synaptic vesicle	19	2.548427
	GO:0005794~Golgi apparatus	127	3.149324

	GO:0015630~microtubule cytoskeleton	85	3.561803
	GO:0070013~intracellular organelle lumen	238	4.426508
	GO:0042995~cell projection	103	5.456561
Molecular Function	GO:0019899~enzyme binding	102	6.27E-04
	GO:0046872~metal ion binding	571	0.006589
	GO:0043169~cation binding	572	0.017693
	GO:0043167~ion binding	578	0.02774
	GO:0051020~GTPase binding	30	0.031498
	GO:0008270~zinc ion binding	334	0.046106
	GO:0031267~small GTPase binding	28	0.054999
	GO:0017016~Ras GTPase binding	25	0.185784
	GO:0005085~guanyl-nucleotide exchange factor activity	34	0.648109
	GO:0046873~metal ion transmembrane transporter activity	61	0.658748
	GO:0046914~transition metal ion binding	384	0.684135
	GO:0008092~cytoskeletal protein binding	85	1.213221
	GO:0008134~transcription factor binding	86	1.358523
	GO:0019904~protein domain specific binding	60	1.425775
	GO:0030695~GTPase regulator activity	70	1.808413
	GO:0016563~transcription activator activity	70	2.672279
	GO:0060589~nucleoside-triphosphatase regulator activity	70	3.241935
	GO:0022843~voltage-gated cation channel activity	31	3.243998
	GO:0005083~small GTPase regulator activity	50	3.456746
	GO:0004674~protein serine/threonine kinase activity	71	5.755474

Supplementary Table 2: Dys-regulated pathways (p-values<0.01) associated with ACM were identified by DAVID.

Category	Term	Count	%	PValue
KEGG_PATHWAY	hsa04010:MAPK signaling pathway	47	2.01	0.001037
	hsa00562:Inositol phosphate metabolism	16	0.68	0.001055
	hsa05414:Dilated cardiomyopathy	21	0.9	0.00447
	hsa04070:Phosphatidylinositol signaling system	18	0.77	0.004708
	hsa04510:Focal adhesion	37	1.58	0.006156
	hsa04020:Calcium signaling pathway	33	1.41	0.007611
	hsa05215:Prostate cancer	19	0.81	0.014472
	hsa05216:Thyroid cancer	9	0.38	0.016108
	hsa05222:Small cell lung cancer	18	0.77	0.017088
	hsa05212:Pancreatic cancer	16	0.68	0.018884
	hsa05223:Non-small cell lung cancer	13	0.56	0.021032
	hsa04210:Apoptosis	18	0.77	0.02368
	hsa00604:Glycosphingolipid biosynthesis	6	0.26	0.024603
	hsa04660:T cell receptor signaling pathway	21	0.9	0.025853
	hsa04810:Regulation of actin cytoskeleton	36	1.54	0.028
	hsa05221:Acute myeloid leukemia	13	0.56	0.035388
	hsa05014:Amyotrophic lateral sclerosis (ALS)	12	0.51	0.042056
	hsa04666:Fc gamma R-mediated phagocytosis	18	0.77	0.050519
	hsa05220:Chronic myeloid leukemia	15	0.64	0.053181
	hsa05211:Renal cell carcinoma	14	0.6	0.063137
	hsa04520:Adherens junction	15	0.64	0.06407
	hsa04912:GnRH signaling pathway	18	0.77	0.064667
	hsa00310:Lysine degradation	10	0.43	0.067665
	hsa05410:Hypertrophic cardiomyopathy (HCM)	16	0.68	0.070304
	hsa04360:Axon guidance	22	0.94	0.076563
	hsa04150:mTOR signaling pathway	11	0.47	0.079271
	hsa05213:Endometrial cancer	11	0.47	0.079271

	hsa04620:Toll-like receptor signaling pathway	18	0.77	0.081297
BIOCARTA	h_ctcfPathway:CTCF: First Multivalent Nuclear Factor	7	0.3	0.041372
	h_fasPathway:FAS signaling pathway (CD95)	9	0.38	0.046127
	h_p38mapkPathway:p38 MAPK Signaling Pathway	9	0.38	0.064165
	h_hdacPathway:Control of skeletal myogenesis by HDAC & calcium/calmodulin-dependent kinase (CaMK)	6	0.26	0.066071
	h_atrbrcaPathway:Role of BRCA1, BRCA2 and ATR in Cancer Susceptibility	7	0.3	0.076547
	h_bard1Pathway:BRCA1-dependent Ub-ligase activity	4	0.17	0.079928
	h_arfPathway:Tumor Suppressor Arf Inhibits Ribosomal Biogenesis	5	0.21	0.084664
